# Comparative sequence analysis of pPATH pathogenicity plasmids in *Pantoea agglomerans* gall-forming bacteria

**DOI:** 10.3389/fpls.2023.1198160

**Published:** 2023-07-31

**Authors:** Naama Geraffi, Priya Gupta, Naama Wagner, Isaac Barash, Tal Pupko, Guido Sessa

**Affiliations:** ^1^ School of Plant Sciences and Food Security, George S. Wise Faculty of Life Sciences, Tel Aviv University, Tel Aviv, Israel; ^2^ The Shmunis School of Biomedicine and Cancer Research, George S. Wise Faculty of Life Sciences, Tel Aviv University, Tel Aviv, Israel

**Keywords:** *Pantoea agglomerans*, sugar beet, gypsophila, type 3 secretion system, type 3 secreted effectors, plasmid, genome assembly, gall-forming

## Abstract

Acquisition of the pathogenicity plasmid pPATH that encodes a type III secretion system (T3SS) and effectors (T3Es) has likely led to the transition of a non-pathogenic bacterium into the tumorigenic pathogen *Pantoea agglomerans*. *P. agglomerans* pv. *gypsophilae* (*Pag*) forms galls on gypsophila (*Gypsophila paniculata*) and triggers immunity on sugar beet (*Beta vulgaris*), while *P. agglomerans* pv. *betae* (*Pab*) causes galls on both gypsophila and sugar beet. Draft sequences of the *Pag* and *Pab* genomes were previously generated using the MiSeq Illumina technology and used to determine partial T3E inventories of *Pab* and *Pag*. Here, we fully assembled the *Pab* and *Pag* genomes following sequencing with PacBio technology and carried out a comparative sequence analysis of the *Pab* and *Pag* pathogenicity plasmids pPATH_pag_ and pPATH_pab_. Assembly of *Pab* and *Pag* genomes revealed a ~4 Mbp chromosome with a 55% GC content, and three and four plasmids in *Pab* and *Pag*, respectively. pPATH_pag_ and pPATH_pab_ share 97% identity within a 74% coverage, and a similar GC content (51%); they are ~156 kb and ~131 kb in size and consist of 198 and 155 coding sequences (CDSs), respectively. In both plasmids, we confirmed the presence of highly similar gene clusters encoding a T3SS, as well as auxin and cytokinins biosynthetic enzymes. Three putative novel T3Es were identified in *Pab* and one in *Pag*. Among T3SS-associated proteins encoded by *Pag* and *Pab*, we identified two novel chaperons of the ShcV and CesT families that are present in both pathovars with high similarity. We also identified insertion sequences (ISs) and transposons (Tns) that may have contributed to the evolution of the two pathovars. These include seven shared IS elements, and three ISs and two transposons unique to *Pab*. Finally, comparative sequence analysis revealed plasmid regions and CDSs that are present only in pPATH_pab_ or in pPATH_pag_. The high similarity and common features of the pPATH plasmids support the hypothesis that the two strains recently evolved into host-specific pathogens.

## 1 Introduction

*Pantoea agglomerans* is a Gram‐negative facultative anaerobic bacterium of the Erwineaceae family (Adeolu et al., 2016). It is widespread in nature and found in association with many plant species as an epiphyte and endophyte (Manulis and Barash, 2003; Sulja et al., 2022). Strains of *P. agglomerans* have evolved into tumorigenic pathogens displaying host specificity on various plants by acquiring a pathogenicity plasmid, which is designated as pPATH. Two *P. agglomerans* pathogenic pathovars can be distinguished: *P. agglomerans* pv. *gypsophilae* (*Pag*), which induces galls on gypsophila and triggers an immune response on sugar beet, and *P. agglomerans* pv. *betae* (*Pab*), which causes galls on both beet and gypsophila (Weinthal et al., 2007; Barash and Manulis-Sasson, 2009). Pathogenicity of both pathovars is dependent on a type III secretion system (T3SS) and effectors (T3Es), and on auxin and cytokinins biosynthetic pathways that are all encoded in the pathogenicity plasmids pPATH_pag_, in *Pag*, and pPATH_pab_, in *Pab* (Manulis and Barash, 2003). The extensively characterized pPATH_pag_ plasmid has a size of ~131 kb and contains a pathogenicity island (PAI) of ~75 kb that harbors genes encoding T3SS structural, regulatory and effector proteins, and plasmid maintenance determinants, and carries multiple insertion sequences (IS) (Lichter et al., 1996; Guo et al., 2002; Weinthal et al., 2007). The PAI structure, composition and location on the plasmid support a recent evolution of pathogenesis (Manulis and Barash, 2003).

The inventory of T3Es in *Pab* and *Pag* bacteria was previously determined based on draft genome sequences in combination with a machine-learning approach and translocation assays into beet roots, where eight and nine plasmid-borne effectors were identified in *Pab* and *Pag* strains, respectively (Nissan et al., 2018). Five of them (DspA/E, HopX2, HopAY1, HopAF1, and HrpK) are in common between *Pag* and *Pab*, and shared with other phytopathogenic bacteria (Lindeberg et al., 2005; Petnicki-Ocwieja et al., 2005; Boureau et al., 2006; Washington et al., 2016; Saint-Vincent et al., 2020). HopD1 was also reported in other bacteria (Block et al., 2014) but it is only present in *Pag*. Conversely, four T3Es (HsvB, HsvG, PthG and PseB) were only identified in *Pag* and *Pab* strains. HsvG and HsvB are putative transcription factors which may contribute to host specificity in gypsophila and beet, respectively (Valinsky et al., 1998; Nissan et al., 2006; Nissan et al., 2012). PthG is present only in *Pag* and triggers an immune response in beet species, while PseB is present only in *Pab* and its function is still unknown (Nissan et al., 2018). The small repertoire and plasmid location of T3Es in the two pathovars are consistent with recent evolution of *P*. *agglomerans* pathogenesis and limited functional redundancy between effectors. Remarkably, transformation of HsvG and PthG or HsvB and PseB was found to convert nonpathogenic bacteria into host-specific gall-forming pathogens on gypsophila and beet, respectively (Nissan et al., 2019).

Draft genome sequences of the *Pab* 4188 and *Pag* 824-1 strains were previously generated using MiSeq second-generation sequencing technology and partially assembled into 79 and 55 contigs for *Pab* and *Pag*, respectively (Nissan et al., 2018). In this study, we employed Pacific Biosciences (PacBio) third-generation sequencing technology, which provides longer reads than MiSeq (Bachall, 2009), to sequence and completely assemble the *Pab* and *Pag* genomes. Comparative sequence analysis of the newly assembled pPATH_pag_ and pPATH_pab_ pathogenicity plasmids identified common and unique genes involved in plasmid housekeeping and bacterial virulence that may have shaped the evolution of the *Pag* and *Pab* pathogenic pathovars.

## 2 Materials and methods

### 2.1 Bacterial strains and growth conditions

The bacterial strains used are *Pantoea agglomerans* pv. *betae* strain 4188 (*Pab*) (Burr et al., 1991) and *Pantoea agglomerans* pv. *gypsophilae* strain 824-1 (*Pag*) (Manulis et al., 1991). These strains were grown at 28°C in Lysogeny Broth (LB) medium supplemented with Rifampicin (100 µg/ml). The same strains were sequenced before (Nissan et al., 2018). The strains used for both sequencing efforts (MiSeq, PacBio) were drawn from the same stock, which was kept frozen in -80°C. Thus, it is unlikely that mutations have accumulated between the two sequencing efforts.

### 2.2 PacBio library construction and DNA sequencing

Bacteria were grown overnight in LB liquid medium, and bacterial genomic DNA was isolated as described by Chen and Kuo (1993). The DNA was sent to Macrogen (Seoul, South Korea) for sequencing. PacBio/single-molecule real-time (SMRT) sequencing was used to sequence the genome of the *Pag* and *Pab* strains. Samples were prepared according to standard instructions for SMRTbell templates for sequencing on the PacBio RS System, and were sequenced using SMRT^®^ sequencing. In *Pag*, the sequencing yielded 82,397 reads (692,414,886 read bases). The read N50 was 12,640 bp and the average read length was 8,748 bp. In *Pab*, the sequencing yielded 81,985 reads (706,785,250 read bases). The read N50 was 12,688 bp and the average read length was 8,906 bp.

### 2.3 Genome assembly and correction

The PacBio reads were used to complete the assembly of the bacterial genomes. These reads are long, and thus allow achieving longer contigs, and in bacterial genomes even the full chromosome sequence. Nevertheless, they are prone to more errors than Illumina reads. In order to obtain a more accurate assembly, previously published draft genome sequences from MiSeq data (Nissan et al., 2018) were used to correct the assembly done using PacBio reads. The PacBio reads were used as input to Canu v1.7 (Koren et al., 2017) to generate the draft complete assembly, with the following parameters: -pacbio-raw corMhapSensitivity=high genomeSize= 5m. The average coverage was assessed by mapping corrected and trimmed reads obtained by Canu v1.7 against the assembly using BWA v0.7.17 (Li and Durbin, 2009; Li and Durbin, 2010), calculating the alignment depth using SAMtools v1.3.3 (Danecek et al., 2021), and the average depth per molecule using awk. Next, we used Circlator (Hunt et al., 2015) to convert the linear contigs into a circular sequence. To run Circlator, the following additional programs were used: BWA v0.7.17 (Li and Durbin, 2009; Li and Durbin, 2010), Prodigal v2.6.3 (Hyatt et al., 2010), Canu v1.7 (Koren et al., 2017), SAMtools v1.3.3 (Danecek et al., 2021), and MUMmer v3.23 (Kurtz et al., 2004). Following this step, we used the abovementioned Illumina reads to polish the assembly using Pilon v1.22 (Walker et al., 2014). To this end, we mapped the Illumina reads to the draft genome using BWA v0.7.17 (Li and Durbin, 2009; Li and Durbin, 2010), converted the output SAM file to BAM file and sorted it using SAMtools v1.3.3 (Danecek et al., 2021), and finally used it to correct the assembly using Pilon with the default parameters values and including –changes to keep track of the corrections done in the assembly. We repeated this process until no further corrections were introduced to the assembly. Two and three rounds were required to fully correct *Pab* and *Pag* assemblies, respectively. The average coverage of the Illumina reads was assessed in the same manner as assessed for the PacBio reads.

### 2.4 Genome annotation and alignment

Genomes were annotated using two different programs: (i) Prokka v1.13.3 (Seemann, 2014) with default parameter values; (ii) RAST: a webserver that was used with default settings (Aziz et al., 2008; Overbeek et al., 2014; Brettin et al., 2015). Finally, ISFinder (Siguier et al., 2006) was used to find and locate ISs and Tns in the plasmids. Whole genome alignment was performed using Mugsy-1.2.2. (Angiuoli and Salzberg, 2011). CDSs were aligned using Emboss Needle global alignment (Rice et al., 2000).

## 3 Results

### 3.1 Assembly of *Pab* and *Pag* genome sequences

Draft genome sequences of the *Pab* 4188 and *Pag* 824-1 strains (~5 Mb) (NCBI accession no. ASM166202v1 and ASM166198v1) were previously generated by MiSeq second-generation sequencing technologies and partially assembled into 79 contigs for *Pab* and 55 for *Pag* (Nissan et al., 2018). In this study, PacBio third-generation sequencing technology, which provides longer reads than MiSeq (Bachall, 2009), was employed to sequence the *Pab* and *Pag* genomes. The newly sequenced data (NCBI accession no.: ASM166202v2 and ASM166198v2), as well as the previously sequenced MiSeq sequencing data, were used to assemble the genome, aiming that the short-read data would correct errors introduced to the assemblies using the long-read data. Both the sequencing data and the final assemblies were deposited to NCBI and can be found under BioProject PRJNA320975.

Assembly of the *Pab* and *Pag* PacBio reads revealed four and five circular contigs respectively, representing the chromosome for each strain, three plasmids for *Pab*, and four plasmids for *Pag* (**Table 1**). The chromosomes have a similar length of ~4 Mb with a 55% GC content and each consists of ~4,000 CDSs. Among the plasmids, the previously identified pPATH pathogenicity plasmids *Pab* and *Pag* (pPATH_pab_ and pPATH_pag_; Manulis and Barash, 2003), have a length of ~156 kb and ~131 kb, respectively, a 51% GC content, and consist of 163 and 138 CDSs, respectively. Two other homologous plasmids were identified in the two pathovars: Plasmid 02 with a length of ∼540 kb in *Pab* and ∼580 kb in *Pag*, and Plasmid 03 with a length of ~180 kb in *Pab* and ∼140 kb in *Pag*. Plasmid 02 and 03 have a GC content ranging between 52% and 54%, and they consist of ~600 and ~200 CDSs, respectively. An additional ~79 kb plasmid, Plasmid 04, was detected in *Pag*. It has a 52% GC content and consists of ~80 CDSs. In a BLASTn search, Plasmid 04 was found to be homologous to plasmid pAR1aD of the *P. agglomerans* strain AR1a (accession no. CP059087) with 67% coverage and 99.8% identity, and to the pEM02 plasmid of *Erwinia* spp. (accession no. LN907829) with 44% coverage and 98% identity.

Table 1Features of *Pab* and *Pag* genomes following sequencing with PacBio, assembly with Canu, polishing with Pilon, and annotation with PGAP.

**Table:** 

*Pab*					
Feature	Chromosome	pPAB02	pPAB03	pPATHpab	
Size (bp)	4,165,783	541,337	178,621	156,057
No. of circular contigs	1	1	1	1
No. of CDSs	3,810	516	156	163
G+C content (%)	55.4	53.4	52.4	50.8
Pac-bio average coverage	39X	34X	21X	37X
Illumina average coverage	606X	614X	660X	1,259X
No. of tRNA genes	77	0	0	0

**Table:** 

*Pag*					
Feature	Chromosome	pPAG02	pPAG03	pPATHpag	pPAG04
Size (bp)	4,098,036	582,658	143,524	131,449	78,538
No. of circular contigs	1	1	1	1	1
No. of CDSs	3,748	572	127	138	83
G+C content (%)	55.3	53.1	53.5	50.7	52
Pac-bio average coverage	41.3X	22.8X	21.9X	31.5X	18.5X
Illumina average coverage	734X	735X	788X	1,372X	1,319X
No. of tRNA genes	77	0	0	0	0

### 3.2 Comparative analysis of the pPATH_pab_ and pPATH_pag_ plasmids

**Figure 1:**
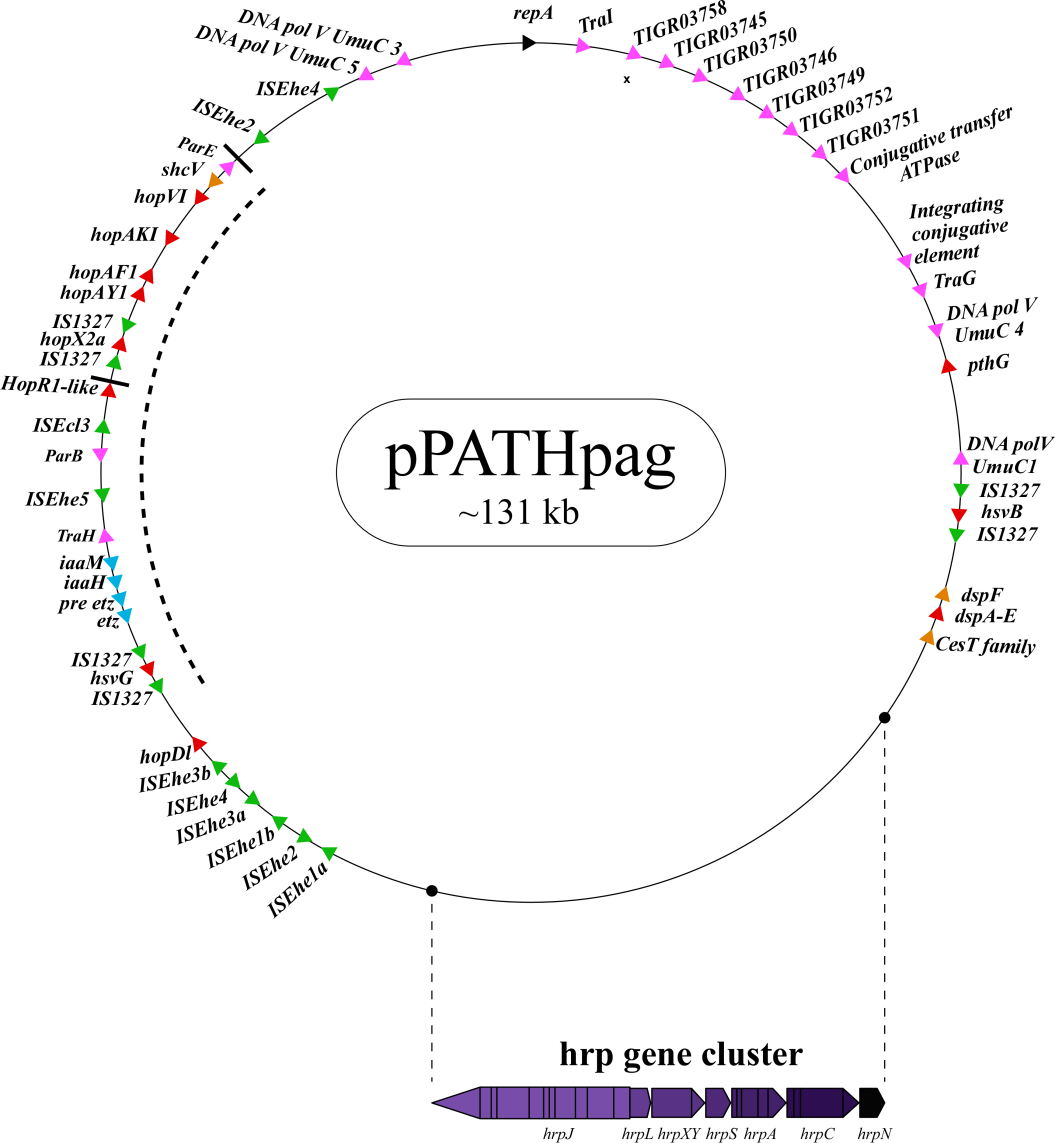
Schematic representation of the pPATH_pag_ plasmid. The plasmid contains the *hrp* gene cluster, genes encoding validated and putative T3SS effector proteins (red), a gene cluster encoding indole-3-acetic acid (IAA) and cytokinins (CK) biosynthetic genes (blue), insertion sequence (IS) elements (green), plasmid maintenance genes (pink), T3C (orange) and the *repA* gene (black). Arrows indicate gene orientation. A cluster of effector genes is marked by thick bars, and an inversion fragment by a dotted line.

**Figure 2:**
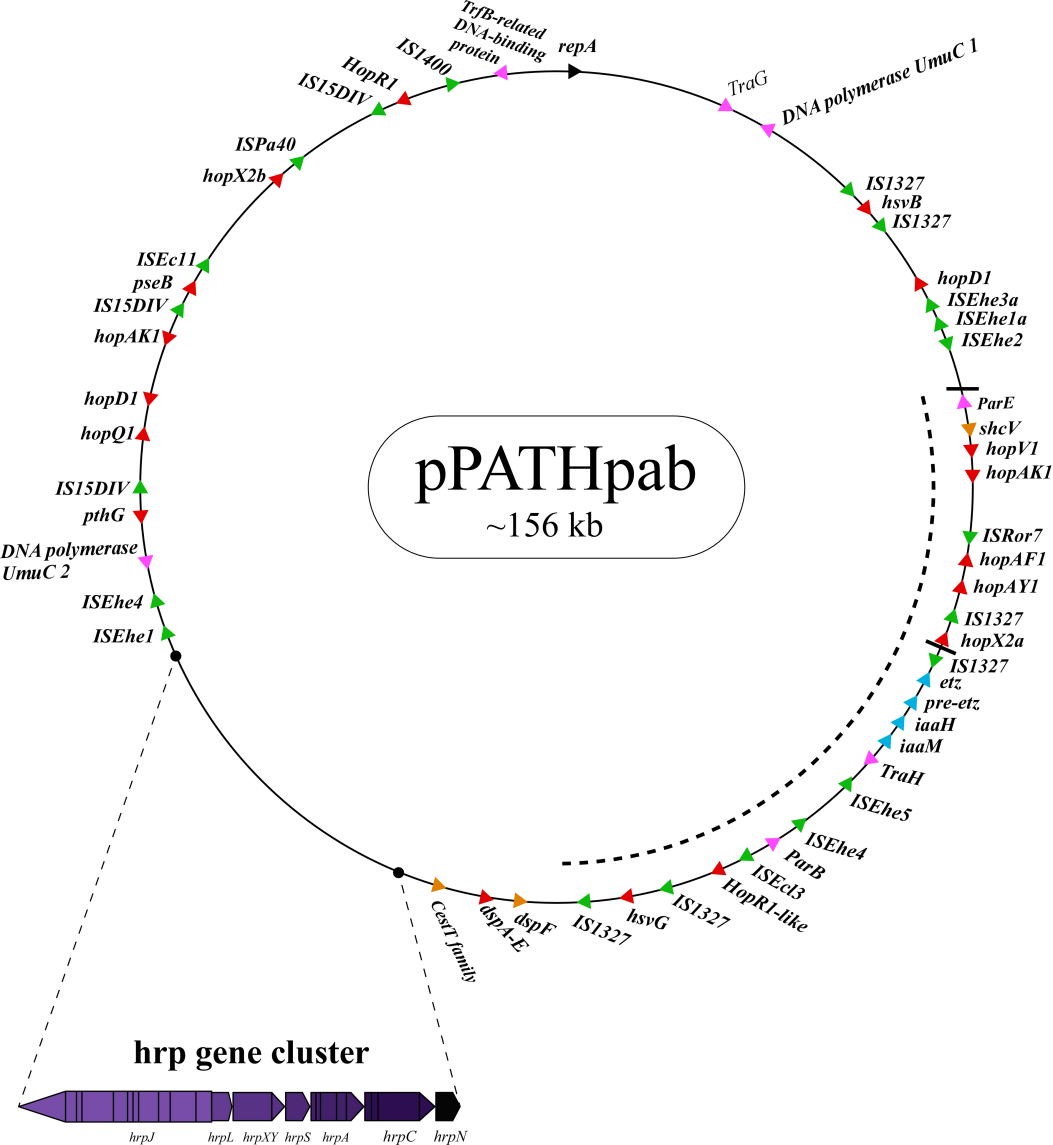
Schematic representation of the pPATH_pab_ plasmid. The plasmid contains the *hrp*\*hrc* gene cluster, genes encoding validated and putative T3SS effector proteins (red), a gene cluster encoding indole-3-acetic acid (IAA) and cytokinins (CK) biosynthetic genes (blue), insertion sequence (IS) elements (green), plasmid maintenance genes (pink), T3C (orange) and the *repA* gene (black). Arrows indicate gene orientation. A cluster of effector genes is marked by thick bars, and an inversion fragment by a dotted line.

Next, detailed comparative analysis was carried out for proteins encoded in the pPATH_pab_ and pPATH_pag_ pathogenicity plasmids (Barash and Manulis-Sasson, 2009). This analysis detected proteins that are involved in plasmid housekeeping and plant pathogenicity, including proteins required for plasmid maintenance, structural and regulatory proteins of the T3SS, T3Es, Type 3 chaperons (T3Cs), harpins, and enzymes of biosynthetic pathways of plant growth hormones. Homologous proteins encoded in the pPATH_pab_ and pPATH_pag_ plasmids were compared and their closest homolog in other bacteria was determined. The obtained data were used to generate an updated map of pPATH_pag_ (**Figure 1**) and the first map of pPATH_pab_ (**Figure 2**).

To examine if other bacterial strains have plasmids with similar structures as observed in pPATH_pab_ and pPATH_pag_, we conducted a small-scale comparative genomics analysis with publicly available *P. agglomerans* genomes. Specifically, we searched for genes related to the T3SS, which in *Pantoea* are known to be encoded on plasmids. We have blasted (tblastn) the protein sequences of the T3SS regulators and components listed in **Tables 2**, **3** versus all the fully assembled genomes of *P. agglomerans* available in NCBI. To consider the presence of each of the components, an E-value lower than 10^-10^ and percentage of identical matches higher than 50% were required. Interestingly, a full cluster was found in one genome – *P. agglomerans* strain DAPP-PG734, on plasmid P2. This cluster was not identified in any other genome. In addition to the T3SS, we also searched for the effectors HsvB and HsvG using tblastn. These effectors were found only on pPATH plasmid of *Pag* and *Pab*. These results suggest that the presence of a T3SS and associated effectors is a derived state that characterizes a few specific strains rather than an ancestral state that characterizes the entire *P. agglomerans* species.

**Table 2:** *Pab* and *Pag* regulatory Hrp proteins.

Protein	^a^ *Pab*\*Pag* Identity (%)	^b^Species with Closest Homolog	Function	Source
HrpY	100	*Erwinia psidii* (86%)	Transcription factor Activates HrpS Acts as response regulator of HrpX	(Wei et al., 2000b; Nizan-Koren et al., 2003)
HrpX	100	*Erwinia mallotivora* (85%)	PAS domain S-box protein Function as sensor	(Wei et al., 2000b; Nizan-Koren et al., 2003)
HrpS	100	*E. mallotivora* (84%)	Transcriptional factor of the NtrC family Activates HrpL	(Nizan-Koren et al., 2003)
HrpL	100	*E. psidii* (82%)	Alternative sigma factor. Activates genes containing “hrp box” promoter	(Nizan-Koren et al., 2003)
HrpT	100	*E. psidii* (69%)	Downregulates T3SS gene expression independent of HrpV	(Ortiz-Martín et al., 2010)
HrpG	100	*E. mallotivora* (68%)	Inhibitor of HrpV; regulates HrpC operon; chaperon-like	(Gazi et al., 2015)
HrpV	100	*Erwinia pyriflorinigrans* (63%)	Interacts with HrpS to diminish the activation of T3SS genes	(Gazi et al., 2015)

^a^Percentage of identity is based on alignment of Pab and Pag protein sequences obtained using BLASTp. ^b^The closest homolog was determined by using the Pag protein sequence as query in BLASTp searches.

**Table 3:** *Pab* and *Pag* structural Hrp\Hrc proteins.

Protein	^a^ *Pab*\*Pag* Identity (%)	^b^Species with Closest Homolog	Activity/Function	Source
HrcC	100	*E. psidii* (85%)	Outer membrane ring subunit	(Portaliou et al., 2016)
HrpF	100	*Erwinia pyrifoliae* (86%)	Stabilizes HrpA prior to formation of pilus interacts with HrpG and downregulates T3SS expression	(Huang et al., 2016)
HrpE	100	*E. mallotivora* (73%)	Stator protein stabilizes HrcN to the membrane in *P. syringae.* Act as pilus subunit in *Xanthomonas*	(Weber and Koebnik, 2006; Portaliou et al., 2016)
HrpD	100	*E. mallotivora* (69%)	ATPase co-factor	(Portaliou et al., 2016)
HrcJ	100	*E. psidii* (85%)	Inner membrane ring lipoprotein	(Portaliou et al., 2016)
HrpB	99	*E. mallotivora* (75%)	Inner rod Positive regulator of virulence pathways	(Genin et al., 1992; Occhialini et al., 2005; Portaliou et al., 2016)
HrpA	100	*E. mallotivora* (80%)	Pilus/Injectisome	(Wei et al., 2000a; Portaliou et al., 2016)
HrpJ	99	*E. mallotivora* (79%)	Gatekeeper subunit Interacts with chaperone-effector complex and prevents effector secretion	(Portaliou et al., 2016)
HrcV	100	*E. mallotivora* (89%)	Export apparatus subunit	(Portaliou et al., 2016)
HrpQ	100	*E. mallotivora* (74.11%)	Inner membrane ring	(Portaliou et al., 2016)
HrcN	100	*E. mallotivora* (89%)	ATPase	(Portaliou et al., 2016)
HrpO	99	*E. tracheiphila* (68%)	Stalk	(Portaliou et al., 2016)
HrcQa	100	*E. psidii* (66%)	Cytoplasmic ring protein	(Portaliou et al., 2016)
HrcQb	100	*E. mallotivora* (69%)	Cytoplasmic ring protein	(Portaliou et al., 2016)
HrcR	100	*E. psidii* (95%)	Export apparatus subunit	(Portaliou et al., 2016)
HrcS	100	*Erwinia tracheiphila* (98%)	Export apparatus subunit	(Portaliou et al., 2016)
HrcT	100	*E. tracheiphila* (85%)	Export apparatus subunit	(Portaliou et al., 2016)
HrcU	100	*E. psidii* (86%)	Export apparatus subunit	(Portaliou et al., 2016)

^a^Percentage of identity is based on alignment of Pab and Pag protein sequences obtained by using BLASTp.

^b^The closest homolog was determined by using the Pag protein sequence as query in BLASTp searches.

#### 3.2.1 Housekeeping proteins

Proteins involved in plasmid maintenance, replication, and transfer were found to be encoded in the pPATH_pab_ and pPATH_pag_ plasmids (**Table 4**). Within this group of proteins is RepA that in *Pseudomonas* was shown to initiate plasmid replication by binding to the origin of replication (Díaz-López et al., 2003; Weinthal et al., 2007), which is yet to be determined in pPATH_pab_ and pPATH_pag_. Several proteins of the partition system that assures equal segregation of chromosome and plasmids were also detected (Bignell and Thomas, 2001; Funnell, 2016). We also found a transcriptional repressor and toxin which are involved in plasmid maintenance. These include a TrfB-related DNA binding protein (transcriptional repressor of genes involved in plasmid inheritance) (Thomas and Smith, 1986), and ParA and ParB partitioning proteins (Bignell and Thomas, 2001; Funnell, 2016). The ParE toxin is predicted to inhibit DNA gyrase to stop replication during stress conditions (Jiang et al., 2002), and to be involved in plasmid maintenance via post segregation killing of plasmid free daughter cells using toxin antitoxin systems (Engelberg-Kulka and Glaser, 1999). ParA is only present in pPATH_pag_, while ParB and ParE are present in both pathovars. In addition, we found two and four copies of polymerase V in *Pab* and *Pag*, respectively. Polymerase V participates in DNA repair (Wang, 2001). Interestingly, proteins involved in the conjugative transfer of integrative conjugative elements (ICEs) are present in pPATH_pag_. ICEs are self-transmissible mobile genetic elements that encode the machinery for conjugation, as well as regulatory systems to control their excision and conjugative transfer (Baltrus et al., 2022; Daveri et al., 2023). They include six integrating conjugative element proteins, a conjugal transfer protein, a conjugal transfer lipoprotein, and a conjugative transfer ATPase. In addition, we found two proteins, TraH (contains an ATP binding motif) and TraG (NTPase), encoded in pPATH of both pathovars, whose homologs in other bacteria participate in pilus synthesis and assembly (Zatyka and Thomas, 1998). Finally, a TraI domain containing protein, which plays a putative function as DNA helicase/relaxase, was detected in pPATH_pag_ in the proximity of RepA and can be a part of relaxosome that facilitates plasmid transfer (Matson and Ragonese, 2005).

**Table 4:** Housekeeping proteins.

Protein	^a^ *Pab*\*Pag* identity (%)	^b^Species with closest homolog	Function	Source
ParB partition protein	100	*Chimaeribacter arupi* (76%)	Helps in plasmid and chromosome partition/DNA binding protein	(Bignell and Thomas, 2001)
ParA family protein	Only *Pag*	*Chimaeribacter arupi* (90%)	Helps in plasmid and chromosome partition/Membrane-associated ATPase	(Bignell and Thomas, 2001)
RepA	100	*Klebsiella pneumoniae* (95%)	Replication initiation protein	(Spiers and Bergquist, 1992)
DNA polymerase V UmuC 1 (152 aa)	100	*Serratia marcescens* (82%)	Involved in translesion DNA synthesis	(Wang, 2001)
DNA polymerase V UmuC 2 (265 aa)	Only *Pab*	*Klebsiella pneumoniae (*95%)	Involved in translesion DNA synthesis	(Wang, 2001)
DNA polymerase V UmuC 3 (40 aa)	Only *Pag*	*Klebsiella pneumoniae* (100%)	Involved in translesion DNA synthesis	(Wang, 2001)
DNA polymerase V UmuC 4 (130aa)	Only *Pag*	*Erwinia oleae* (86%)	Involved in translesion DNA synthesis	(Wang, 2001)
DNA polymerase V UmuC 5 (340 aa)	Only *Pag*	*Erwinia rhapontici* (98%)	Involved in translesion DNA synthesis	(Wang, 2001)
TraH	100	*Salmonella enterica* (86%) *E. mallotivora* (86%)	Pilus assembly	(Zatyka and Thomas, 1998)
TraG	100	*Klebsiella pneumoniae* (100%)	Pilus assembly	(Zatyka and Thomas, 1998)
Integrating conjugative element	Only *Pag*	*Klebsiella pneumoniae* (99%)	Unknown function	(Baltrus et al., 2022)
TIGR03758 family integrating conjugative element	Only *Pag*	*Dryocola clanedunensis* (53%)	Unknown function	(Baltrus et al., 2022)
TIGR03745 family integrating conjugative element	Only *Pag*	*Klebsiella oxytoca* (60%)	virB2/iceB2 (Precursor for conjugative pilus)	(Daveri et al., 2023)
TIGR03750 family conjugal transfer protein	Only *Pag*	*Kalamiella piersonii* (67%)	Unknown function	(Baltrus et al., 2022)
TIGR03746 family integrating conjugative element	Only *Pag*	*Erwinia rhapontici* (88%)	Unknown function	(Baltrus et al., 2022)
TIGR03749 family integrating conjugative element	Only *Pag*	*Duffyella gerundensis* (88%)	Unknown function	(Baltrus et al., 2022)
TIGR03752 family integrating conjugative element	Only *Pag*	*Duffyella gerundensis* (90%)	virB10/part of Type IV component	(Voth et al., 2012)
TIGR03751 family conjugal transfer lipoprotein	Only *Pag*	*Duffyella gerundensis* (96%)	Outer membrane protein	(Daveri et al., 2023)
TraI	Only *Pag*	*Erwinia* *rhapontici* (94%)	Putative DNA helicase	(Matson and Ragonese, 2005)
Conjugative transfer ATPase	Only *Pag*	*Duffyella gerundensis* (93%)	Unknown	(Baltrus et al., 2022)
TrfB-related DNA-binding protein	99	*Duffyella* *gerundensis* (93%)	Transcriptional repressor	(Thomas and Smith, 1986)
ParE family toxin	100	*Pseudomonas* sp. *T1.Ur* (85%)	Inhibit DNA gyrase	(Jiang et al., 2002; Kamruzzaman and Iredell, 2019)

^a^Percentage of identity is based on alignment of Pab and Pag protein sequences obtained by using BLASTp.

^b^The closest homolog was determined by using the Pag protein sequence as query in BLASTp searches.

#### 3.2.2 Structural and regulatory proteins of the type III secretion system

**Figure 3:**
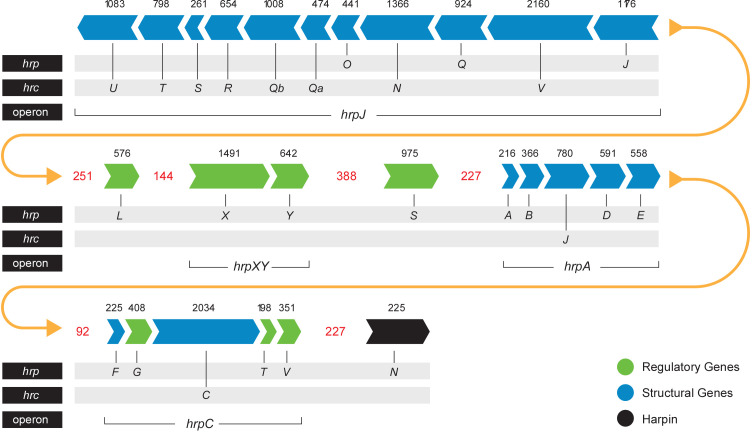
*Pab* and *Pag hrp*\*hrc* gene cluster. Arrows indicate gene orientation. Black numbers denote the gene size (base pairs). Red numbers denote the distance (base pairs) between operons\genes. Yellow arrows represent the continuity of the gene cluster.

The T3SS is a syringe-like structure that delivers effector proteins inside the plant cell (Galán and Collmer, 1999). It is a complex of proteins encoded by *hrp* (hypersensitive response and pathogenicity) and *hrc* (hypersensitive response and conserved) genes (Alfano and Collmer, 1997). Structure and function of the *Pag* T3SS were extensively characterized in previous studies (Nizan et al., 1997; Mor et al., 2001). Here, we identified and compared structural and regulatory T3SS proteins of pPATH_pab_ and pPATH_pag_ and determined their closest homologs in other bacteria (**Tables 2**, **3**). All Hrp\Hrc proteins are identical in the two pathovars. They display 63%-98% sequence similarity to proteins in different *Erwinia* spp., most commonly *Erwinia mallotivora* and *Erwinia psidii*. As schematically shown in **Figure 3**, the *hrp\hrc* gene cluster consists of four operons: *hrpJ, hrpA, hrpC, hrpXY*, and three single genes: *hrpL*, *hrpS* and *hrpN*. The genetic arrangement of these operons was found to be the same as in *Pag* (Mor et al., 2001). Operons *hrpJ, hrpA* and *hrpC* mainly encode T3SS structural components, while the *hrpXY* operon encodes regulatory proteins (**Tables 2**, **3**). The *hrpJ* operon is the largest and consists of 11 genes (*hrpJ*, *hrcV*, *hrpQ*, *hrcN*, *hrpO*, *hrcQa*, *hrcQb*, *hrcR*, *hrcS*, *hrcT* and *hrcU*), all encoding structural proteins of the T3SS basal body, except HrcN which is an ATPase and HrpJ that acts as a gatekeeper protein that regulates translocator and effector secretion (Portaliou et al., 2016). *hrpA* is a smaller operon and consists of five genes (*hrpA*, *hrpB*, *hrcJ*, *hrpD* and *hrpE*) encoding pilus/injectisome (HrpA, HrpB and HrcJ) components, an ATPase cofactor (HrpD) and a stator protein (HrpE) to stabilize HrcN. The last structural operon is *hrpC* consisting of five genes (*hrpF*, *hrpG*, *hrcC*, *hrpT* and *hrpV*) with different functions. Homologs of *hrpT*, *hrpV* and *hrpG* in *Pseudomonas syringae* and *Erwinia amylovora* were shown to have a regulatory role (Ortiz-Martín et al., 2010; Gazi et al., 2015). They act in concert to control *hrp/hrc* gene expression which should be coupled with the assembly and function of the T3SS under inducing condition (Ortiz-Martín et al., 2010). The *hrpXY* two-gene operon together with hrpS and hrpL is responsible for regulation of T3SS genes that contain a hrp box in their promoter (**Tables 2**, **3**).

#### 3.2.3 Type III effectors

T3Es are secreted through the T3SS directly inside the plant cell and manipulate host cellular processes to promote bacterial growth in the apoplast (Macho, 2016). Previous reports identified nine effectors in *Pag* (HsvG, HsvB, DspA/E, HopAY1, HopX2, HopAF1, HrpK, PthG, and HopD1) and eight in *Pab* (HsvG, HsvB, PseB, DspA/E, HopAY1, HopX2, HopAF1 and HrpK) (Nissan et al., 2018). Truncated forms of PthG and HopD1 were found in *Pab*, and a truncated HopAY1 was found in *Pag*. In addition, homologs of HopV1 and HopR1 effectors, which are known to be functional in other bacteria (Wei et al., 2007), were found, but their translocation was not assessed by secretion assays (Nissan et al., 2018). Notably, in our analysis, HrpK was not retrieved in any of the pathovar assemblies. The composition of the *Pab* and *Pag* T3E pools was further refined by the identification of three new candidate effectors in pPATH_pab_ and one in pPATH_pag_. One of these candidate T3Es was named HopR1-like, based on its similarity to HopR1 of *Pab* (47%). HopR1-like is present and identical in the two pathovars and represents a new member of the AvrE-family of T3Es displaying 96% similarity to a transducer protein in *E. psidii* (**Table 5**). Two additional newly identified candidate effectors present in *Pab* are HopQ1 and HopX2b. HopQ1 displays high sequence similarity (98%) to HopQ1 of *P. syringae* pv. *tomato* DC3000 and to XopQ of *Xanthomonas euvesicatoria* (63%) (Giska et al., 2013; Teper et al., 2014). HopX2a (previously reported as HopX2) is present in both pathovars. Another CDS (HopX2b) which is more closely related (98%) to HopX2 of *P. syringae*, was found only in *Pab* (**Table 5**). HopX2b displays 70% identity to HopX2a and they both belong to XopE/AvrPphe family. However, translocation of HopR1-like, HopX2b and HopQ1 into plant cells and their contribution to bacterial virulence is yet to be determined.

**Table 5:** *Pab* and *Pag* T3Es.

Effector	^a^ *Pab*\*Pag* Identity (%)	^b^Species with closest homolog	Putative function\target	^c^Translocation	Source
HsvB	91	none	Transcription factor\gall elicitation in beet	+	(Nissan et al., 2006)
HsvG	98	none	Transcription factor\gall elicitation in gypsophila	+	(Nissan et al., 2006)
DspA/E	100	*E. mallotivora* (73%)	Cell-death inducer	+	(Boureau et al., 2006)
HopAF1	98	*Pseudomonas amygdali* (94%)	Inhibits ethylene biosynthesis	+	(Washington et al., 2016)
HopX2a (327 aa)	100	*P. syringae* (80%)	Cysteine protease	+	(Nissan et al., 2018)
HopR1-like	100	*E. psidii* (96%)	Possibly cytoplasmic arginine transducer	ND	(Storch et al., 1999)
*PthG	28	*P. syringae* pv. *coryli* (88%)	Gall elicitation in gypsophila\HR in beet	+	(Ezra et al., 2004)
*HopD1	57	*P. syringae* (93%)	Suppression of effector‐triggered immunity	+	(Block et al., 2014)
*HopAY1	67	*P. syringae* (85%)	Cysteine‐type endopeptidase activity	+	(Nissan et al., 2018)
HopQ1	Only *Pab*	*P. syringae* pv. *tomato* DC3000 (98%)	14-3-3 protein binding	ND	(Giska et al., 2013)
HopX2b (353 aa)	Only *Pab*	*P. syringae* (98%)	Cysteine protease (70% similarity to HopX2a)	ND	
PseB	Only *Pab*	none	Gall elicitation in beet	+	(Nissan et al., 2019)
^○^HopR1	Only *Pab*	*Pseudomonas caricapapayae* (96%)	Possibly suppresses callose formation	–	(Kvitko et al., 2009)
^○^HopV1	100	*Pseudomonas coronafaciens* (89%)	Contributes to virulence but not to growth	–	(Wei et al., 2007)

^a^Percentage of identity based on an alignment of Pab and Pag protein sequences obtained by using pairwise sequence alignment (https://www.ebi.ac.uk/Tools/psa/emboss_needle/)

^b^The closest homolog for proteins present in both pathovars was determined by using the Pag protein sequence as a query in a BLASTp search.

^c^Translocation ability as reported by Nissan et al. (2018).

*Truncated or possibly truncated effectors in one pathovar.

^○^Proteins with effector-like features whose translocation was tested, but not detected by Nissan et al. (2018).

ND, Not determined.

T3Es of the two pathovars, either in full-length or truncated, display a high degree of sequence similarity (91%-100%). The majority of them display high sequence similarity to effectors of other bacteria, mainly of *Pseudomonas* spp. (73%-97%) (**Table 5**). Remarkably, in this study we found a homolog in *P. syringae* pv. *coryli* (87.91%) for PthG that, along with HsvB, HsvG and PseB, has not been previously detected in any other bacteria. Most of the effector genes are distributed throughout the pPATH_pab_ and pPATH_pag_ plasmids, with the exception of a gene cluster including the HopV1, HopAF1, HopAY1, and HopX2a effector genes, and the HopAKI harpin (**Figures 1**, **2**). Putative functions of the effectors are listed in **Table 5**.

#### 3.2.4 Harpins

Harpins represent a class of proteins secreted through the T3SS that facilitate translocation of T3Es into plant cells (Li et al., 2019a). Our analysis confirmed the presence of the previously reported harpins HrpN and HopAK1 in both pathovars (Nissan et al., 2018), and identified an additional homolog of HopAK1 in pPATH_pab_ (HopAK1-1). Sequence comparison revealed that HrpN of *Pab* and *Pag* are almost identical (99%), while HopAK1 homologs of the two pathovars display 91% similarity. Closest homologs of HopAK1 and HrpN were found in *P. syringae* (86%) and *E. psidii* (65%), respectively. In terms of location of the genes within the pPATH plasmids, *hrpN* is at the edge of the *hrp*\*hrc* cluster in both pathovars, while *hopAK1* is located within a cluster of effector genes (**Figures 1**, **2**). *Pab hopAK1-1* encodes a harpin, which has its closest homolog in *P. syringae* (64%) and is located upstream of the *PseB* effector gene.

#### 3.2.5 Type 3 chaperons

T3Cs are small (15-20 kDa), cytoplasmic, and acidic proteins that play roles in T3Es secretion, such as prevention of T3E premature aggregation and cytoplasmic proteolysis (Lohou et al., 2013). Our analysis detected three T3Cs that are encoded in both pPATH plasmids: DspF, ShcV and CesT (**Table 6**). DspF was previously reported to be present in *Pag* (Mor et al., 2001) and shares relatively high sequence similarity to DspF of *E. piriflorinigrans* (74%). In *E. amylovora* it was shown to facilitate translocation of the DspA/E T3E by interacting with its N-terminus through a predicted β-sheet helix-binding groove (Gaudriault et al., 2002; Triplett et al., 2009). ShcV displays the highest similarity to its *Pseudomonas coronafaciens* homolog (88%). ShcV was reported to interact with and assist the translocation of HopPtoV effector in *P. syringae*. This effector-chaperon interaction is also supported by the genomic location of these two proteins: the CDS for the ShcV T3C and the HopPtoV T3E are adjacent to each other (Wehling et al., 2004). In pPATH_pab_ and pPATH_pag_, ShcV and DspF are encoded by CDS adjacent to the *HopV1* and *DspA/E* T3E genes, respectively (**Figures 1**, **2**) in support of the hypothesis that they play a function as chaperones of the encoded T3Es. An additional T3C encoded in both *Pab* and *Pag* is a member of the CesT family of chaperons that were shown to assist in the recruitment of multiple T3Es to the T3SS (Thomas et al., 2005). It shares a relatively low sequence similarity to a protein in *E. psidii* (53%) and its location upstream to the *DspA/E* CDS suggests its involvement in folding and\or secretion of this effector.

**Table 6:** Type III chaperones.

Chaperon	^a^ *Pab*\*Pag* identity (%)	^b^Species with closest homolog	Putative function\target	Source
ShcV	100	*Pseudomonas coronafaciens* (88%)	HopPtoV secretion and translocation	(Wehling et al., 2004)
DspF	99	*E. piriflorinigrans* (74%)	DspE stability and secretion	(Triplett et al., 2010)
CesT family	99	*E. psidii* (53%)	Multi effector chaperon	(Thomas et al., 2005)

^a^Percentage of identity based on an alignment of *Pab* and *Pag* protein sequences obtained by using globular alignment emboss needle.

^b^The closest homolog was determined by using the *Pag* protein sequence as query in BLASTp search.

#### 3.2.6 Biosynthetic enzymes of plant hormones

Galls formation may be caused by interference of the bacteria with the hormone balance of the plant, in particular with the ratio between auxin and cytokinin concentrations. We identified four plant hormone biosynthetic genes (*iaaM*, *iaaH*, *etz* and *pre-etz*) in both the pPATH plasmids, as previously reported for pPATH_pag_ (**Table 7**) (Lichter et al., 1995; Manulis et al., 1998). IaaM and IaaH are enzymes participating in auxin synthesis through the indole-3-acetamide pathway (Morris, 1986). The operon for cytokinin biosynthesis consists of two genes: *pre-etz* and *etz*. The function of *pre-etz* is unknown, while *etz* encodes the enzyme isopentenyl transferase (Guo et al., 2001). The similarity of these genes in the two pathovars is high (96%-97%), and all enzymes, except pre-Etz, are very similar to homologs in *Erwinia* spp. No putative homologs have been found for pre-Etz. All four genes are clustered together in the pPATH_pag_ and pPATH_pab_ plasmids (**Figures 1**, **2**).

**Table 7:** Biosynthetic enzymes of plant hormones.

Enzyme	^a^ *Pab*\*Pag* identity (%)	^b^Species with closest homolog	Hormone synthesized
Tryptophan 2-monooxygenase iaaM	96	Bacteria symbiont BFo1 of *Frankliniella occidentalis* (99%)	Auxin
Indoleacetamide hydrolase iaaH	96	Bacteria symbiont BFo1 of *Frankliniella occidentalis* (98%)	Auxin
Pre-Etz	96	–	Cytokinin
Etz	97	*E. tracheiphila* (57%)	Cytokinin

^a^Percentage of identity is based on alignment of *Pab* and *Pag* protein sequences obtained by using BLASTp.

^b^The closest homolog was determined by using the *Pag* protein sequence as query in BLASTp searches.

#### 3.2.7 Mobile transposable elements

TEs, including ISs and Tns, are major determinants in the evolution of pathogenic bacteria (Siguier et al., 2014; Nicolas et al., 2015). Tns differ from ISs because in addition to the transposase, they carry passenger/cargo genes, which are not involved in catalysis or regulation of the TE movement (Siguier et al., 2014). ISs belong to diverse families and groups based on the type of transposase, number of CDSs, size, conserved terminal base pairs at the end, number of base pairs present in direct repeats produced at the target site after transposition, and mechanism of transposition (Mahillon and Chandler, 1998). Previous studies detected the presence of ISEhe*1*, ISEhe*2*, ISEhe*3*, ISEhe*4*, ISEhe*5*, IS*1327* (six copies) in pPATH_pag_ (Lichter et al., 1996; Guo et al., 2002). Information about the presence of ISs and Tns in *Pab* was not reported earlier.

In this study, we used the ISFinder tool to retrieve TE sequences in pPATH_pab_ and pPATH_pag_, and sequences with the highest significance were analyzed for their location in the plasmid and number of copies. This analysis identified ten types of ISs and two Tns (ISPa*40* and ISRor*7*) in pPATH_pab_ and seven ISs in pPATH_pag_. Seven of all the identified ISs are common to both pathovars (ISEhe*1*, ISEhe*2*, ISEhe*3*, ISEhe*4*, ISEhe*5*, IS*1327*, ISEcl*3*) and belong to diverse IS families: IS1, IS3, IS5, IS6 and IS630. Exclusively present in *Pab* are the ISs IS*1400*, ISEcl*1* and IS*15DIV* and the Tns ISRor*7* and ISPa*40*. In *Pag* we found an additional copy of ISEhe*2* and ISEhe*4*, and new IS ISEcl*3* (**Table 8**). The presence of such diverse ISs indicates massive horizontal gene transfer (HGT) (Barash and Manulis-Sasson, 2009).

**Table 8:** Insertional sequences and transposons present in pPATH_pab_ and pPATH_pag_.

IS/Tn	IS Family	Group	Size range (bp)	^*^DR (bp)	Ends	^#^No of CDSs	^@^Chemistry of the enzyme	Mechanism	Origin	Copies in *Pab*	Copies in *Pag*	Comments	Accession No (ISfinder)
ISEhe*5*	IS*1*		740-1180	8-9	GGnnnTG	2	DDE	Copy and paste Co-integrate	*P. agglomerans*	1	1		AY665723
ISEhe*4*	IS*3*	IS*407*	1100-1400	4	TG	2	DDE	Copy and paste	*P. agglomerans*	2	2		AF324174
ISEhe*3*	IS*3*	IS*51*	1000-1400	3-4	TG	2	DDE	Copy and paste	*P. agglomerans*	1	1	Disrupted (in 2 parts in *Pag*; only first part in *Pab*)	AF327445
ISEhe*2*	IS*5*	IS*427*	800-1000	2-4	Ga/g	2	DDE	–	*P. agglomerans*	1	2		AF327444
IS132*7*	IS*6*	-	700-900	8	GG	1	DDE	Co-integrate	*P. agglomerans*	6	6		X87144
ISEhe*1*	IS*630*	-	1000-1400	2		1 or 2	DDE	–	*P. agglomerans*	1	1	Disrupted (in 2 parts in *Pag*; only first part in *Pab*)	AF326767
IS*1400*	IS*3*	IS*407*	1100-1400	4	TG	2	DDE	Copy and paste	*Y. enterocolitica*	1	–	Host- *Yersinia pseudotuberculosis* IP32938 *Yersinia enterocolitica* O5 *Yersinia enterocolitica* O13 *Yersinia enterocolitica* Ye 8081 *Yersinia pseudotuberculosis* IP32954	X94452
ISEcl*1*	IS*3*	IS*2*	1300-1400	5	TG	2	DDE	Copy and paste	*E. cloacae*	1	–		AF342826
ISEcl*3*	IS*5*	IS*903*	950-1150	9	TG	1	DDE	–	*Enterobacter cloacae*	1	1		AY780889
IS*15DIV*	IS*6*	–	700-900	8	GG	1	DDE	Co-integrate	*Salmonella typhimurium*	3	–		X13616
ISRor*7*	Tn*3*		3150	0		1	DDE	–	*Raoultella ornithinolytica*	1	–	Host-*Raoultella ornithinolytica* 170602815 plasmid p602815-NR	MN310380
ISPa*40*/TnPa*40*	Tn*3*		6592	0		5	DDE	–	*P. aeruginosa*	1	–	Host-*Pseudomonas aeruginosa* DK2	–

All the information was obtained by using ISFinder. All ISs have terminal inverted repeats. ^*^DR-Direct repeats formed after transposition at the target site.

^#^CDS-coding sequence for transposase enzyme except ISPa40 which has passenger and accessory genes in addition to transposase.

^@^DDE represents the common acidic triad of aspartate (D), aspartate, glutamate (E); presumed to be part of the active site of the transposase.

Homologs of ISPa*40*, IS*1400* and IS*15DIV* were found in plant and animal pathogenic bacteria, such as *P. aeruginosa*, *Yersinia enterocolitica* and *Salmonella typhimurium*. In contrast, ISEhe*1*, *3*, *5* and IS*1327*, display very little similarity to proteins in other bacteria (Lichter et al., 1996; Guo et al., 2002). As previously reported by Guo et al. (2002), ISEhe*3* and ISEhe*1* are separated into two parts in *Pag* due to the insertion of ISEhe*4* and ISEhe*2*, respectively. Conversely in *Pab*, we found only the first fragment of ISEhe*3* and ISEhe*1*. It is likely that the second fragment of ISEhe*3* and ISEhe*1* has been lost during evolution of pPATH_pab_ (**Figures 1**, **2**). All ISs are quite dispersed throughout the pPATH plasmid in both pathovars, though in pPATH_pag_ there is a typical clustering of ISEhe*1*-*4* downstream to the T3SS cluster (**Figures 1**, **2**).

#### 3.2.8 Unique CDSs

**Figure 4:**
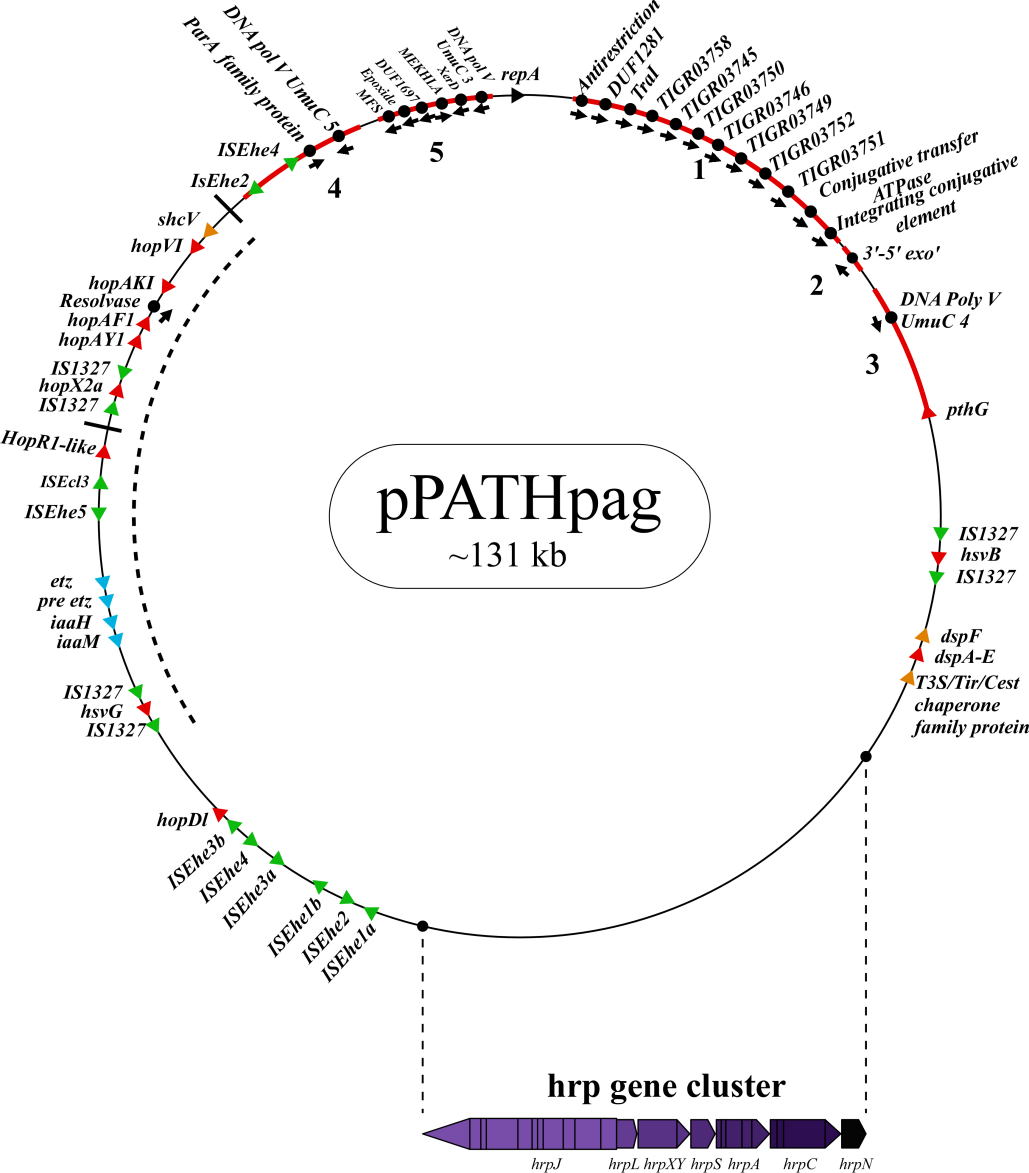
Unique sequences of the pPATH_pag_ plasmid. Sequences that are present in pPATH_pag_, but not in pPATH_pab_, are marked in red and numbered from 1 to 5. Genes located on these unique sequences and present in pPATH_pag_, but not in pPATH_pab_, are indicated with black dots. Color code for arrows for rest of the genes are similar to **Figure 1**.

To investigate differences between pPATH_pab_ and pPATH_pag_, we aligned the two plasmids and analyzed the CDSs in all the unaligned fragments. Sequences that are present in pPATH_pag,_ but not in pPATH_pab,_ are indicated in **Figure 4** (fragments 1-5). Three unique sequences are located downstream to the *repA* gene: the first (~15 kb) contains a CDS encoding an antirestriction protein, DUF1281 with unknown function, six integrating conjugative element protein, TIGR03750 family conjugal transfer protein, and TIGR03751 family conjugal transfer lipoprotein (**Figure 4**, fragment 1). The second fragment (261 bp) encodes a 3’-5’ exonuclease (**Figure 4**, fragment 2). The third one (1,524 bp) contains a CDS that encodes a polymerase V (**Figure 4**, fragment 3). Fragment 4 (2,131 bp) contains CDSs encoding two ISs (ISEhe2 and ISEhe4), a membrane-associated ATPase (ParA family protein), and two polymerase V (**Figure 4**). Fragment 5 of the unique area (5,930 bp) ends closely to *repA*. It contains seven CDSs that are unique to *Pag* and encode: MFS (major facilitator superfamily) transporter, which has a role in resistance to toxic compounds, epoxide hydrolase, DUF1697, a protein containing a MEKHLA domain, tyrosine recombinase XerC that is involved in transposition, polymerase V, and peptidase (**Figure 4**). The vast majority of these CDSs display high sequence similarity to genes present in genomes of *Erwinia* spp. (85%-96%) (**Table 9**).

**Table 9:** Unique CDS in *Pag*.

Protein	^a^Species with closest homolog	Category	Putative function\target	Source
zincin-like metallopeptidase domain-containing protein/ Antirestriction protein	*Duffyella gerundensis* (97%)	General	Protects the DNA from host endonucleases during conjugation	(González-Montes et al., 2020)
DUF1281	*Klebsiella pneumoniae* (99%)	General	Unidentified protein	
TraI	*Erwinia* *rhapontici* (94%)	Plasmid mobility	Putative DNA helicase	(Matson and Ragonese, 2005)
Integrating conjugative element	*Klebsiella pneumoniae* (99%)	Present on integrative conjugative elements (ICEs)	Unknown	(Baltrus et al., 2022)
TIGR03758 family integrating conjugative element	*Dryocola clanedunensis* (53%)	Present on integrative conjugative elements (ICEs)	Unknown	(Baltrus et al., 2022)
TIGR03745 family integrating conjugative element	*Klebsiella oxytoca* (60%)	Present on integrative conjugative elements (ICEs)	virB2/iceB2 (Precursor for conjugative pilus)	(Daveri et al., 2023)
TIGR03750 family conjugal transfer protein	*Kalamiella piersonii* (67%)	Present on integrative conjugative elements (ICEs)	Unknown	(Baltrus et al., 2022)
TIGR03746 family integrating conjugative element	*Erwinia rhapontici* (88%)	Present on integrative conjugative elements (ICEs)	Unknown	(Baltrus et al., 2022)
TIGR03749 family integrating conjugative element	*Duffyella gerundensis* (88%)	Present on integrative conjugative elements (ICEs)	Unknown	(Baltrus et al., 2022)
TIGR03752 family integrating conjugative element	*Duffyella gerundensis* (90%)	Present on integrative conjugative elements (ICEs)	virB10/part of Type IV component	(Daveri et al., 2023)
TIGR03751 family conjugal transfer lipoprotein	*Duffyella gerundensis* (96%)	Present on integrative conjugative elements (ICEs)	Outer membrane protein	(Daveri et al., 2023)
Conjugative transfer ATPase	*Duffyella gerundensis* (93%)	Present on integrative conjugative elements (ICEs)	Unknown	(Baltrus et al., 2022)
3’-5’ exonuclease	*E. hormaechei* (89%)	General	DNA proofreading	(Shevelev and Hübscher, 2002)
DNA polymerase V UmuC 4 (130aa)	*Erwinia oleae* (86%)	DNA repair	Involved in translesion DNA synthesis	(Wang, 2001)
ParA family protein	*Chimaeribacter arupi* (90%)	Plasmid partition	Helps in plasmid and chromosome partition/Membrane-associated ATPase	(Bignell and Thomas, 2001)
Error-prone, lesion bypass DNA polymerase; UmuC 5 (340 aa)	*Erwinia rhapontici* (98%)	DNA repair	Involved in translesion DNA synthesis	(Wang, 2001)
DNA polymerase V; UmuC 3 (40aa)	*Klebsiella pneumoniae* (100%)	DNA repair	Involved in translesion DNA synthesis	(Wang, 2001)
XerD/site specific integrase	*E. rhapontici* (92%)	Mobility accessory	site-specific recombinase Functions in circular chromosome separation	(Subramanya et al., 1997)
MFS Transporter	*P. seleniipraecipitans* (55%)	Resistance	Resistance to various toxic compounds and antibiotics	(Vela-Corcía et al., 2019)
Epoxide hydrolase	*P. seleniipraecipitans* (75%)	General	Detoxification of xenobiotics, regulation of signalling pathways and mediation of virulence Hydrolyze epoxides	(Morisseau and Hammock, 2013; Archelas et al., 2016; Bahl et al., 2016; Stojanovski et al., 2020)
MKHLA domain containing protein	*Erwinia* sp. *AG740 (*63%)	General	Putative bacterial sensor histidine kinases	(Mukherjee and Bürglin, 2006)
DUF1697	*Microvirga* sp. *3-52 (*66%)	General	Unidentified protein	
Resolvase	*P. amygdali* pv *mori* (66%)	Transposon mobility	Resolving the cointegrate (Fusion of donor having Tn3 family transposons and target DNA molecule) in site-specific recombination	(Nicolas et al., 2015)

^a^Species with closest homolog were determined by using BLASTp.

**Figure 5:**
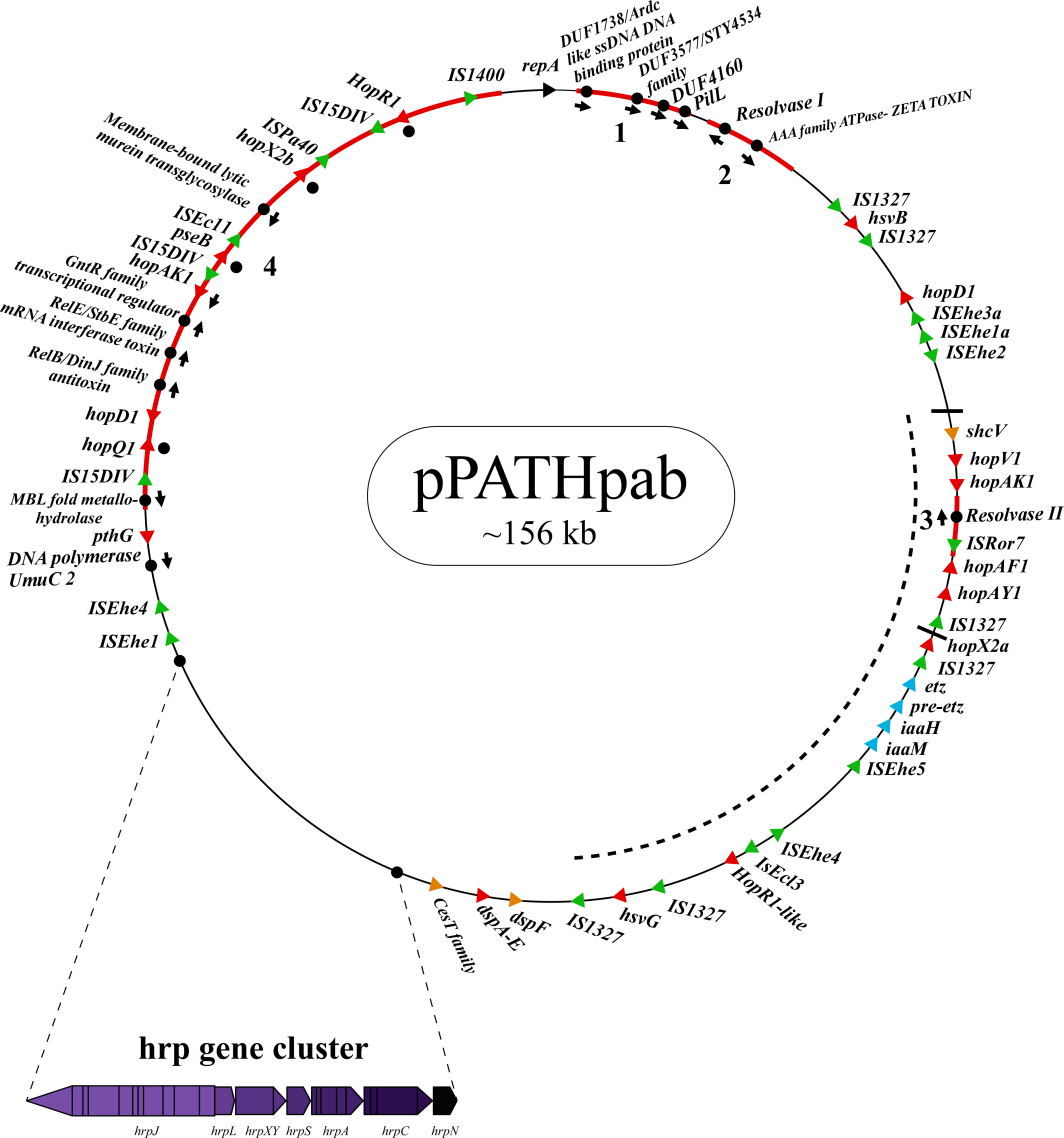
Unique sequences of the pPATH_pab_ plasmid. Sequences that are present in pPATH_pab_, but not in pPATH_pag_, are marked in red and numbered from 1 to 4. Genes located on these unique sequences and present in pPATH_pab_, but not in pPATH_pag_, are indicated with black dots. Color code for arrows for rest of the genes are similar to **Figure 2**.

Sequences that are present in pPATH_pab_ but not in pPATH_pag_ are indicated in **Figure 5**. There are two consecutive unique sequences in the first ~16 kb downstream to repA (**Figure 5**, fragments 1 and 2). Fragment 1 contains four CDSs encoding ArdC-like ssDNA-binding domain-containing protein/DUF1738 (antirestriction protein), STY4534 family ICE replication protein/DUF3577 (unknown function), DUF4160 (unknown function) and a pilL protein involved in pilus assembly. Fragment 2 encodes a resolvase I gene involved in recombination processes, and an ATPase gene involved in the zeta toxin\antitoxin system. An additional unique sequence of ~5,400 bp is located within the T3E cluster (**Figure 4**, fragment 3); it includes a resolvase II gene and the transposon ISRor*7*, which belongs to the Tn3 family. There is also a ~20 kb unique sequence upstream to repA (**Figure 5**, fragment 4) that includes a copy of HopD1 and HopAKI, and CDSs encoding four effectors present only in pPATH_pab_ (HopQ1, PseB, HopX2b and HopR1). In addition, throughout this region, there are the ISs IS*15DIV* (three copies), ISEcl*1*, IS*1400* and the Tn ISPa*40*. Other unique CDSs within fragment 4 encode proteins involved in type II toxin\antitoxin two components system, which enhances bacteria fitness, antibiotics resistance and maintenance. A CDS next to the truncated PthG encoding polymerase is also unique to *Pab* (**Table 10**).

**Table 10:** Unique CDS in *Pab*.

Protein	^a^Species with closest homolog	Category	Putative function\target	Source
ArdC-like ssDNA-binding domain-containing protein/DUF1738	*Klebsiella pneumoniae* (98%)	Antirestriction protein	Exported during conjugation to recipient cell and protects the DNA from host endonucleases	(González-Montes et al., 2020)
STY4534 family ICE replication protein/DUF3577	*Duffyella gerundensis* (98%)	–	Unknown	(Seth-Smith et al., 2012)
DUF4160	*Klebsiella pneumoniae* (99%)	General	Unknown	
PilL	*Duffyella gerundensis* (98%)	Type IV	Pilus biosynthesis	(Srimanote et al., 2002)
Resolvase - recombinase family protein 1	*Duffyella gerundensis* (96%)	Transposon mobility	Resolving the cointegrate (Fusion of donor having Tn3 family transposons and target DNA molecule) in site-specific recombination	(Nicolas et al., 2015)
AAA family ATPase - ZETA TOXIN	*Curtobacterium plantarum* (69%)	Toxin\anti-toxin system	Targets cell wall formation Arrests growth in an ATP dependent manner	(Jaén-Luchoro et al., 2017)
Resolvase - recombinase family protein 2	*Klebsiella aerogenes* (98%)	Transposon mobility	Resolving the cointegrate (Fusion of donor having Tn3 family transposons and target DNA molecule) in site-specific recombination	(Nicolas et al., 2015)
HopAKI02	*P. syringae* (86%)	Harpin	Pectate lyase	(Kvitko et al., 2007)
HopX2b	*P. syringae* (98%)	Effector	Cysteine proteases	This study
HopQ1	*P. syringae* pv. *tomato* DC3000 (98%)	Effector	14-3-3 protein binding	(Giska et al., 2013)
HopR1	*P. caricapapayae* (96%)	Effector	Possibly suppresses callose formation	(Kvitko et al., 2009)
PseB	–	Effector	Gall elicitation in *Beta vulgaris*	(Nissan et al., 2019)
Membrane-bound lytic murein transglycosylase	*E. psidii* (97%)	Cell wall recycling	Murein-degrading enzyme	(Lee et al., 2013)
GntR family transcriptional regulator	*Pseudomonas* sp. *ES3-33* (55%)	Transcription factor	Involved in many biological processes like cell motility, glucose metabolism, bacterial resistance, pathogenesis expression of multiple sugar transporter and biofilm formation	(Suvorova et al., 2015; Li et al., 2019b; Liu et al., 2021)
RelB/DinJ family antitoxin	*Erwinia persicina* (78%)	Type II toxin-antitoxin system	Antitoxin	(Kamruzzaman and Iredell, 2019)
RelE/StbE family mRNA interferase toxin	*Cronobacter dublinensis* (86%)	Type II toxin-antitoxin system	mRNA cleavage Inhibits translation	(Keren et al., 2004)
MBL fold metallo-hydrolase	*Salmonella enterica* (64%)	Resistance	Resistance to β-lactam antibiotics	(Pettinati et al., 2016)
DNA polymerase V UmuC 2 (265 aa)	*Klebsiella pneumoniae (*95%)	DNA repair	Involved in translesion DNA synthesis	(Wang, 2001)

^a^Species with closest homolog were determined using BLASTp.

## 4 Discussion

Sequencing of the *Pag* and *Pab* genomes by PacBio technology allowed their complete assembly and disclosed the structure and composition of the pPATH_pab_ and pPATH_pag_ pathogenicity plasmids. Sequence analysis of pPATH_pab_ and pPATH_pag_ allows to formulate hypotheses about their evolutionary origin. The high similarity (97%) between pPATH_pab_ and pPATH_pag_ supports the notion that these plasmids evolved from a common ancestor plasmid. CDSs of the ~20 kb *hrp*\*hrc* gene cluster, which is highly conserved in *Pab* and *Pag* (>99% identity), display high similarity to *hrp*\*hrc* genes of *Erwinia* spp. This suggests that the ancestor *P*. *agglomerans* strain, which were possibly non-pathogenic, may have acquired the *hrp*\*hrc* gene cluster from a pathogenic *Erwinia* strain and thereby turned into a new pathogenic strain. In support of this hypothesis, copies of the T3E *DspA/E* are located in *Pag* and *Pab* at the edge of the *hrp*\*hrc* cluster, as similarly observed in *Erwinia* spp. (Siamer et al., 2011), and it is likely that *DspA/E* has been transferred from *Erwinia* to *P*. *agglomerans* along with the *hrp*\*hrc* cluster. Recently, a T3SS has also been reported in endophytic *P. agglomerans* DAPP‐PG 734 and, *P. agglomerans* BAV 2934 but it is distantly related to *Pab* and *Pag* T3SS suggesting different origin of T3SS in different *Pantoea* strains (Moretti et al., 2021; Sulja et al., 2022). It is possible that pPATH was introduced into a *P*. *agglomerans* population by a conjugative or mobilizable plasmid. *P*. *agglomerans* may have acquired the entire pPATH plasmid or the PAI was incorporated in a pre-existing plasmid (Barash and Manulis-Sasson, 2009). In either one of these cases, horizontal gene transfer (HGT) appears as a major evolutionary force that drove pPATH generation. Large mobile elements, such as Tns and ISs, are key players in HGT (Nicolas et al., 2015). The wide genetic interchange between *P*. *agglomerans* and other bacterial strains manifests itself in the large repertoire of IS elements occurring in pPATH_pab_ and pPATH_pag_, and in the presence of T3E genes common to other phytopathogenic bacteria, and particularly widespread among *P. syringae* pathovars (Guo et al., 2002; Manulis and Barash, 2003).

Several lines of evidence indicate that *P*. *agglomerans* pathogenic strains are in an early stage of evolution. First, *P. agglomerans* pathovars have their T3SS gene cluster and effector genes in a plasmid, which suggests that the pPATH plasmids have been acquired recently, and the PAI has not been yet incorporated in the *P*. *agglomerans* chromosome, as observed in other pathogens (Hacker et al., 1997). In addition, comparison between corresponding plasmids of the two pathovars revealed a high identity (96%-97%) and similarity coverage (73%-74%) suggesting that pPATH, plasmid 02 and plasmid 03 were all present in the common ancestor strain before its splitting into two distinct pathovars. Finally, the repertoire of T3Es of the two pathovar is limited as compared to other pathogens. Based on our refined analysis, seven effectors are present in both pathovars (HsvG, HsvB, DspA/E, HopX2a, HopAF1, HopV1 and HopR1-like). In addition, HopD1 and PthG are present only in *Pag*, while PseB, HopQ1, HopAY1, HopR1 and HopX2b are exclusive to *Pab* and are located in a region of ~20 kb that is unique to pPATH_pab_ (**Figure 3**).

Introduction of pathoadaptive mutations represents an important mechanism that may contribute to evolution of a new pathogen (Sokurenko et al., 1999; Bartoli et al., 2016). In support of the involvement of pathoadaptive mutations in the evolution of *Pag* and *Pab*, truncated variants of T3Es are present in the two pathovars: HopAY1 is truncated in *Pag*, while PthG and HopD1 are truncated in *Pab*. These genes acquired mutations that interrupted their CDSs, possibily contributing to the formation of the two distinct pathovars. Truncation of these effectors may have allowed bacteria to escape recognition by newly appeared resistance proteins of the host plant. Generation of PthG in *Pag* may be the result of pathoadaptive changes that occurred randomly and were preserved due to their beneficial effect. One possible scenario is that *Pab* evolved from *Pag* by a genetic modification that resulted in truncation of the PthG CDS and evasion of beet recognition and immunity (Ezra et al., 2000; Manulis and Barash, 2003; Ezra et al., 2004). Typically, these are mutations causing a functional modification or elimination of genes that confer enhanced pathogenicity to the bacteria (Sokurenko et al., 1999).

A 20 kb region which is perfectly mirrored in pPATH_pab_ and pPATH_pag_ is present in the two plasmids. This segment includes the cluster of plant hormone biosynthetic genes, the clustered effector genes, the *hsvG* gene, and the *hopR1-like* candidate effector gene. The inversion must have occurred sometime after the splitting into two pathovars and could have happened spontaneously or due to a replication-transcription conflict that resulted in DNA rearrangement (Merrikh et al., 2012; Merrikh and Merrikh, 2018). It has been known that head-on orientation genes can be beneficial to the bacteria due to their high mutation frequency (Merrikh and Merrikh, 2018). Altogether, we conclude that genetic rearrangements and mutations in the ancestor pathogenic plasmid supposedly shaped pPATH_pag_ and pPATH_pab_ resulting in the generation of two pathogenic strains with different host specificities.

## Data availability statement

The datasets presented in this study can be found in online repositories. The names of the repository/repositories and accession number(s) can be found below: https://www.ncbi.nlm.nih.gov/, BioProject PRJNA320975.

## Author contributions

Conceptualization, GS, IB and TP. Software, TP and NW. Formal Analysis, NG, PG and NW. Investigation, NG and PG. Data Curation, NG and PG. Writing – Original Draft Preparation, NG, PG, GS and IB. Writing – Review and Editing, TP and NW. Supervision, GS, TP and IB. Project Administration, GS. Funding Acquisition, GS and IB. All authors contributed to the article and approved the submitted version.

## In Memoriam

This paper is dedicated to Guido-Sessa (19642023), a scientist, a mentor, and a friend.
